# Motion Analysis Technologies for ACL Injury Prevention: From Laboratory Assessment to Field-Based Clinical Screening

**DOI:** 10.3390/jcm15124686

**Published:** 2026-06-17

**Authors:** Abdulmajeed Alfayyadh

**Affiliations:** Department of Physical Therapy and Health Rehabilitation, College of Applied Medical Sciences, Jouf University, Sakaka 72388, Saudi Arabia; abalfayyadh@ju.edu.sa

**Keywords:** ACL injury prevention, motion capture, marker-less pose estimation, biomechanical screening, machine learning, return-to-sport, field-based assessment, drone systems, wearable sensors

## Abstract

Anterior cruciate ligament (ACL) injuries remain a leading cause of morbidity in athletic populations, with 70–80% occurring through non-contact mechanisms driven by biomechanical risk factors including knee valgus (>10°), low knee flexion (<30°), tibial internal rotation (>20°), and loading asymmetry (>15°), yet implementation of evidence-based neuromuscular training (which reduces injury risk by 50–70%) remains limited due to barriers in identifying at-risk individuals through accessible field-based screening. This narrative review synthesizes motion analysis technologies spanning laboratory-based optical systems (marker-based), wearable inertial measurement units (IMUs), computer vision and marker-less pose estimation, force plate and pressure-sensitive insole systems, and integrated drone-based field assessment platforms to address this critical gap. We present a three-tier clinical screening framework that progresses from basic anthropometric and single-plane video analysis to multi-modal biomechanical assessment using real-time kinematic feedback. As an illustrative example of emerging field-deployable technology, an integrated drone-based motion capture and smart insole system combining 4K video capture, AI-driven 3D motion reconstruction, and plantar pressure mapping is described to demonstrate how laboratory-quality biomechanical assessment can be achieved in ecologically valid field settings. This evidence-based review addresses current gaps between laboratory research and practical field deployment, with emphasis on cost-effectiveness, accessibility, and clinical utility for ACL injury prevention in diverse sporting environments.

## 1. Introduction

Anterior cruciate ligament (ACL) injuries are among the most common and economically burdensome orthopedic injuries in athletic populations, with incidence ranging from 0.07 to 0.24 per 1000 athletic exposures [1,2]. Approximately 70–80% of ACL injuries occur through non-contact mechanisms involving identifiable biomechanical risk factors, principally knee valgus (>10°), low knee flexion at landing (<30°), tibial internal rotation (>20°), and inter-limb loading asymmetry (>15%) [3,4,5,6,7,8,9,10,11,12,13,14,15,16,17]. These represent approximate reference values derived predominantly from prospective cohort studies of collegiate athletes during drop-landing and cutting tasks; specific thresholds may vary by sport, population, and assessment context. It should be noted that while early prospective studies identified knee valgus as a candidate biomechanical risk marker, subsequent prospective investigations have reported inconsistent predictive validity [13,18,19], with some studies finding weak or non-significant associations after controlling for confounders such as sex, sport, and limb dominance [5]. Knee valgus should therefore be interpreted as one component of a multi-factorial risk profile rather than a standalone injury predictor. Female athletes sustain ACL injuries at approximately 2–3 times the rate of male athletes in comparable sports, driven by sex-specific differences in neuromuscular control and lower-extremity kinematics [5,7]. Targeted neuromuscular training programs addressing these modifiable factors reduce ACL injury incidence by 50–70% across diverse athletic populations [5,7].

Despite the efficacy of these interventions, widespread implementation remains limited by the absence of accessible, validated methods for identifying at-risk individuals at scale. Current clinical practice relies predominantly on subjective visual assessment and cost-prohibitive laboratory biomechanical testing, creating a treatment gap in which high-risk athletes proceed through competitive seasons without targeted intervention. Translating laboratory-based biomechanical assessment to practical field-based screening has been further impeded by the technological constraints of traditional optical motion capture systems, which require controlled indoor environments and capital investment of $150,000–$500,000 or more [7].

Recent advances in computer vision, wearable inertial sensors, and deep learning-based marker-less pose estimation have created new possibilities for scalable, cost-effective biomechanical screening. This manuscript systematically reviews motion capture technologies spanning laboratory-based optical systems, inertial measurement units, computer vision pose estimation, and integrated field-deployable platforms. We synthesize validation data, examine machine learning architectures for automated injury risk classification, and present a practical three-tier clinical screening framework designed for progressive complexity and accessibility. As a representative example of emerging field-deployable technology, we describe an integrated drone-based motion capture and smart insole system that combines multi-modal sensor data with AI-driven analysis to enable comprehensive biomechanical assessment in ecologically valid field settings (Section 9).

## 2. Review Methodology

This manuscript is a narrative review conducted to provide a comprehensive critical synthesis of motion analysis technologies relevant to ACL injury prevention, spanning laboratory-based biomechanical systems, wearable inertial sensors, computer vision and marker-less pose estimation, force measurement devices, and integrated field-deployable platforms. A narrative review format was selected in preference to a systematic review given the multi-domain scope of the topic, which encompasses biomechanics, sensor engineering, computer vision, and machine learning, domains that do not share a common primary outcome measure or study design amenable to pooled quantitative synthesis.

A structured literature search was conducted across five electronic databases: PubMed/MEDLINE, Scopus, Web of Science, IEEE Xplore, and SPORTDiscus. The search covered publications from January 2000 to December 2024. Search terms were developed around three thematic domains and combined using Boolean operators: (1) ACL injury and biomechanics, including terms such as “anterior cruciate ligament,” “ACL injury prevention,” “knee biomechanics,” “knee valgus,” and “neuromuscular risk factors”; (2) motion analysis technologies, including “motion capture,” “optical motion capture,” “inertial measurement unit,” “wearable sensors,” “pose estimation,” “marker-less motion capture,” “force plate,” and “pressure insole”; and (3) artificial intelligence and clinical screening, including “machine learning,” “deep learning,” “injury risk classification,” and “clinical screening framework.” Seminal foundational studies published before 2000 were included where directly relevant to establishing the scientific basis for a concept.

Studies were included if they: (1) reported original validation data for a motion analysis technology; (2) investigated biomechanical risk factors for ACL injury in human participants; (3) evaluated machine learning or AI methods for biomechanical classification; or (4) described clinical screening frameworks for ACL injury risk. Studies were excluded if they were: (1) single-case reports; (2) conference abstracts without full-text peer-reviewed publication; (3) non-English-language publications; or (4) studies focused exclusively on post-surgical outcomes without relevance to screening or prevention technology. Reference lists of identified articles were hand-searched for additional relevant studies not captured by electronic search. Final study selection was based on relevance to the review objectives and quality of reported methodology, assessed independently by the author.

During the preparation of this manuscript, Claude (Anthropic, San Francisco, CA, USA), an AI-based tool, was used in a limited capacity as a reference management aid to help organize and compile citations. All references were subsequently verified manually by the author. The author takes full responsibility for the accuracy and integrity of the content of this manuscript.

## 3. Motion Capture Technologies: Optical Marker-Based Systems

### 3.1. Principles and Components of Optical Motion Capture

Optical motion capture (mocap) systems form the current gold standard for three-dimensional (3D) kinematic measurement in biomechanical research. These systems use multiple infrared (IR) cameras positioned around a calibrated capture volume to track the three-dimensional positions of retroreflective markers attached to anatomical landmarks. By triangulating marker positions across cameras, the system reconstructs detailed skeletal kinematics in real time.

The fundamental components include: (1) multiple synchronized infrared cameras (typically 6–16 units for laboratory settings), (2) infrared light sources integrated with each camera, (3) retroreflective spherical markers (4–14 mm diameter), (4) calibration tools (wands, boards) for spatial reference frame establishment, and (5) processing software that identifies markers, reconstructs 3D positions, and computes joint angles using anthropometric models. Leading commercial systems include Vicon (Oxford, UK), Qualisys (Gothenburg, Sweden), and OptiTrack (Corvallis, OR, USA).

### 3.2. System Specifications and Measurement Accuracy

Professional-grade optical mocap systems (exemplified by Vicon systems) typically operate at 100–400 Hz sampling frequency with spatial accuracy of ±0.5–2.0 mm in the capture volume, depending on camera resolution and calibration rigor [20]. A comprehensive 12-camera Vicon system cost approximately $200,000–$400,000 USD in 2023, plus annual licensing and support fees [20]. For comparison, smaller research-grade systems (6–8 cameras) cost $80,000–$150,000, while basic systems for teaching purposes operate at $30,000–$50,000 [20].

The measurement accuracy of optical mocap has been extensively validated. Cappozzo et al. (1995) [21] performed systematic evaluation of marker-based 3D reconstruction accuracy, examining errors from camera calibration, marker identification algorithms, and 3D triangulation. Under controlled conditions, positional accuracy of individual markers was ±1–3 mm within the capture volume, with accuracy degrading toward the periphery. Della Croce et al. (1999) extended these evaluations by examining the propagation of marker position errors through kinematic angle calculations, finding that errors of ±2 mm in marker position resulted in joint angle errors of ±2–4° for ankle and knee measurements [22].

### 3.3. Soft Tissue Artifact and Technical Limitations

A major technical limitation of marker-based systems is soft tissue artifact (STA), the movement of skin and subcutaneous tissue relative to the underlying bone during dynamic movement. This causes retroreflective markers to move relative to bone landmarks, introducing systematic and random errors in kinematic calculations. Leardini et al. (2005) [23] quantified soft tissue artifacts at the knee by implanting bone pins in cadaver specimens and comparing marker-derived kinematics to bone-based measurements. Peak soft tissue artifact ranged from 5 to 15 mm depending on anatomical location and movement type, with greatest errors at the femur and smallest at the tibia. Peters and Baker (2008) documented that STA could contribute ±5–10° errors in calculated knee flexion angles during dynamic movements, necessitating careful marker placement and redundant anatomical landmarks [24]. These errors carry direct clinical implications: given that ACL injury risk thresholds for knee valgus and flexion angle are typically defined within narrow ranges (e.g., valgus > 10°, flexion < 30°), STA-induced errors of ±5–10° can meaningfully influence risk classification decisions. STA is particularly problematic at the knee, where the thigh segment contains substantial soft tissue mass, making femoral marker displacement the dominant source of error. Clinicians interpreting marker-based kinematic data for ACL injury risk assessment should therefore apply conservative interpretation margins, treat borderline threshold values with caution, and consider STA compensation algorithms or bone-anchored reference measurements where high precision is required.

Additional limitations of optical mocap in field settings include: (1) requirement for calibrated indoor laboratory space with controlled lighting, (2) vulnerability to infrared interference from sunlight and environmental IR sources, (3) marker occlusion during rapid or complex movements, (4) time-intensive marker placement and system calibration, and (5) cost-prohibitive equipment investment. These constraints create a “laboratory dependency” that limits large-scale screening applications.

### 3.4. Anatomical Landmark Calibration and Kinematic Computation

Optical mocap kinematic accuracy depends critically on precise anatomical landmark identification and marker placement protocol adherence. The International Society of Biomechanics (ISB) has published standardized protocols for placement of anatomical markers at bony landmarks including the anterior superior iliac spine, posterior superior iliac spine, lateral femoral condyle, lateral malleolus, and other key anatomical sites [7,17]. Cappozzo et al. (1995) established foundational ISB marker placement recommendations based on anatomical palpation cadaver studies, defining optimal marker positions for subsequent inverse kinematic calculations [21].

Joint angles are calculated using anatomical coordinate systems defined by marker triads (sets of three non-collinear markers). Della Croce et al. (1999) formalized the mathematical approach for defining anatomical reference frames and computing joint angles using Euler angle conventions, providing the kinematic calculation methodology that is now standard across all mocap systems [22]. For the knee, a typical calculation defines the femoral frame using markers on the femur and hip, defines the tibial frame using markers on the tibia and ankle, and computes flexion/extension, valgus/varus, and rotation angles from these coordinate system orientations.

## 4. Wearable Inertial Measurement Units (IMUs)

### 4.1. IMU Technology Fundamentals

Inertial measurement units (IMUs) represent a fundamentally different technological approach to motion capture, employing miniaturized accelerometers, gyroscopes, and magnetometers to quantify body segment orientation and acceleration. IMUs measure linear acceleration (via accelerometers) and angular velocity (via gyroscopes) in three axes, from which body orientation can be estimated through sensor fusion algorithms combining multiple sensor inputs.

The Xsens MVN (Movella, The Netherlands) system exemplifies professional-grade IMU-based mocap. The Xsens system consists of 17 small sensor modules (approximately 50 mm × 30 mm × 20 mm) positioned at anatomical locations including the sternum, lower back, pelvis, upper and lower arms, upper and lower legs, and feet [7,17]. Each module contains a 3-axis accelerometer (±16 g range), 3-axis gyroscope (±2000°/s range), and 3-axis magnetometer, sampling at 240 Hz with wireless transmission to a central processing unit [25,26]. The system is calibrated through a brief (~30–60 s) neutral standing pose (“N-pose”), during which the user stands in a standardized anatomical position to establish gravitational reference frames and magnetic declination values specific to the local environment [27,28,29].

### 4.2. Xsens System Performance and Validation

Xsens systems have been extensively validated against optical mocap gold standards. Xsens technical specifications claim ±0.2% accuracy for body position in a 9 m × 9 m capture volume and real-time wireless transmission with <50 ms latency [7,17]. Independent validation studies provide quantitative performance metrics. Lebel et al. (2017) compared Xsens MVN to a Vicon optical mocap system in 15 healthy subjects performing walking, running, and lateral cutting tasks [27]. Pearson correlation coefficients for knee flexion angle were r = 0.89 across all movement tasks, with root-mean-square error (RMSE) = 3.8° ± 1.2° [27]. For hip flexion/extension, correlations were r = 0.91 with RMSE = 3.1° ± 0.9° [27]. This represents substantially greater accuracy than earlier-generation IMU systems and establishes Xsens as a validated alternative to optical mocap for many applications.

Schepers et al. (2007) examined the critical role of IMU calibration procedures, comparing results from various calibration protocols in the Xsens system [28]. N-pose calibration (standard 30–60 s neutral pose) produced knee angle errors of ±3–4°, while longer calibration protocols incorporating multiple poses reduced errors to ±2–3°, highlighting the importance of standardized calibration procedures for clinical consistency [28].

### 4.3. Advantages and Limitations of IMU-Based Systems

IMU systems offer substantial advantages over optical mocap: (1) portability, with wireless, battery-operated units suitable for field deployment; (2) no occlusion vulnerability, as motion data are recorded continuously without the risk of marker loss that affects optical systems during sustained or complex movements; (3) no lighting requirements, independent of environmental IR or sunlight; (4) real-time data streaming to mobile devices or analysis platforms; and (5) lower capital cost ($30,000–$80,000 for research-grade Xsens systems compared to $200,000+ for optical systems) [7,17].

However, IMU limitations include: (1) drift in gyroscope integration over extended periods, necessitating periodic drift correction; (2) sensitivity to local magnetic field disturbances from ferrous materials and electronic equipment, affecting magnetometer-based orientation estimation; (3) lower spatial resolution than optical mocap for fine anatomical landmark detail; (4) vendor-specific sensor fusion algorithms that can reduce transparency and reproducibility; and (5) requirement for anatomical calibration before each use session.

### 4.4. Complementary Use: IMU and Optical Mocap Integration

In contemporary research settings, IMUs and optical mocap are often used synergistically rather than as competing technologies. Lebel et al. (2017) demonstrated that integration of Xsens IMU data with optical mocap markers in the same capture volume provides complementary information: optical markers provide gold-standard 3D positioning and reduce gyroscope drift, while IMU accelerometer and gyroscope data provide high-frequency dynamic information and improve robustness during temporary marker occlusions [27]. This multi-modal approach is particularly valuable for comprehensive dynamic assessment during high-intensity athletic movements.

### 4.5. Overview of Additional IMU Manufacturers and Systems

While the Xsens MVN system represents the most extensively validated full-body IMU platform in sports biomechanics research, several other commercially available systems are widely used in peer-reviewed biomechanical, clinical gait, and ACL-related investigations. The APDM Opal V2R system (Clario, Portland, OR, USA) employs up to 24 wirelessly synchronized sensors sampling at 20–800 Hz (default 128 Hz) and has been validated against optical motion capture across a range of athletic and clinical tasks, demonstrating acceptable agreement for lower-extremity kinematics [30,31]. The Noraxon myoMOTION and its successor Ultium Motion (Noraxon USA, Inc., Scottsdale, AZ, USA) support up to 16 sensors at up to 200 Hz and have been specifically validated for change-of-direction and jump-landing tasks directly relevant to ACL injury assessment, yielding cross-correlation coefficients exceeding 0.88 in the sagittal plane [32]. The Delsys Trigno Avanti system (Delsys Inc., Natick, MA, USA) is notable for its concurrent acquisition of surface electromyography and 9-degrees-of-freedom inertial data within a single wearable sensor, enabling simultaneous kinematic and neuromuscular assessment that is particularly valuable in ACL rehabilitation research [33]. The Vicon Blue Trident sensor (Vicon Motion Systems/IMeasureU, Oxford, UK) offers dual-range accelerometry (±16 g and ±200 g simultaneously) at sampling rates up to 1125 Hz, with native hardware synchronization to Vicon optical systems, making it uniquely suited to hybrid laboratory–field assessment protocols and return-to-sport load monitoring [34]. Finally, the Shimmer3 IMU platform (Shimmer Sensing, Dublin, Ireland) provides a modular, low-cost alternative (approximately USD $300–800 per sensor) that has been applied in ACL gait classification studies and is especially relevant in resource-limited clinical and field settings [35]. Across all IMU systems, a consistent limitation for ACL-specific applications is reduced accuracy in the frontal and transverse planes—particularly for knee abduction/adduction—where root-mean-square errors typically exceed those reported for sagittal-plane kinematics, underscoring the importance of selecting and validating systems against task-specific biomechanical benchmarks.

## 5. Computer Vision and Marker-Less Pose Estimation

### 5.1. Deep Learning Foundations for Pose Estimation

Marker-less pose estimation, the inference of human body kinematics directly from standard video without physical markers, has been revolutionized by advances in deep neural networks. Goodfellow et al. (2016) provided foundational exposition of convolutional neural networks (CNNs), recurrent neural networks (RNNs), and generative models, establishing the theoretical and mathematical framework for deep learning-based computer vision [36]. CNNs are particularly well-suited to pose estimation because their convolutional layers inherently capture spatial hierarchies and are translation-invariant, enabling robust detection of body landmarks across image locations and scales.

### 5.2. OpenPose: Multi-Person 2D Pose Estimation

OpenPose (Carnegie Mellon University) is an open-source library for multi-person 2D pose estimation that has become widely adopted in sports biomechanics and clinical research. Cao et al. (2021) presented the OpenPose architecture, which employs a two-branch CNN design: the first branch detects confidence maps (heatmaps) for each anatomical keypoint (joint location), while the second branch predicts part affinity fields (spatial relationships between connected joints) that enable robust association of keypoints into coherent body poses [37,38,39,40]. OpenPose detects 25 anatomical keypoints including head, shoulders, elbows, wrists, hips, knees, and ankles, enabling computation of multi-planar joint angles. The system operates at 30 frames per second on standard laptop CPU hardware without GPU acceleration, making it highly accessible for field-based applications [37,38,39,40].

### 5.3. Validation of Marker-Less Pose Systems

Direct validation of marker-less pose estimation against optical mocap gold standards is essential for clinical adoption. Insafudin et al. (2023) conducted comparative testing of OpenPose against Vicon optical mocap in 20 subjects performing landing and cutting tasks [41]. Pearson correlations for knee flexion angle were r = 0.88–0.95 across subjects, with root-mean-square error (RMSE) = 3–6° depending on anatomical joint and movement task [41]. Hip flexion/extension correlations were r = 0.89–0.93, with RMSE = 4–7° [41]. These validation metrics suggest that OpenPose can provide approximate lower-extremity kinematic assessment within ±3–6° of optical mocap gold standards. However, the clinical acceptability of this accuracy is context-dependent and should not be assumed universally. Errors of 3–6° may be sufficient for gross population-level screening, but become clinically meaningful when risk classification depends on narrow angular thresholds; for example, a 6° error relative to a 10° knee valgus risk cutoff could produce false-positive or false-negative risk classifications with direct consequences for clinical decision-making. Clinicians and researchers adopting marker-less pose estimation systems should therefore explicitly consider the measurement uncertainty relative to their specific risk thresholds, and treat borderline classifications with appropriate caution pending further prospective validation.

MediaPipe validation against optical mocap has received less direct attention in the peer-reviewed literature. Comparable computer vision systems (e.g., Kinect depth camera) have been validated against optical mocap. Colyer et al. (2014) evaluated Kinect v2 marker-less pose estimation against optical mocap in controlled laboratory settings with subjects performing sports movements [42]. 2D spatial projection errors (the measuring discrepancy between the detected and true joint location in the video image plane) were <5 cm at standard filming distances of 5 m, which translates to angular accuracy of ±3–4° for lower-extremity joint angles [42]. While not direct MediaPipe validation, this work suggests that lightweight computer vision systems can achieve clinical-grade accuracy when camera distance and angle are standardized.

### 5.4. Three-Dimensional Convolutional Neural Networks (3D CNNs)

Extending 2D pose estimation to full 3D reconstruction from single-camera views requires additional neural network architectures that model temporal information across video frames. Three-dimensional CNNs (3D CNNs) are convolutional networks that apply convolutions not only across spatial (x, y) dimensions but also across the temporal (t) dimension, treating video as a 4D tensor (x, y, t, channels).

Previous studies showed that 3D CNN applications in sports video analysis, demonstrating that 3D convolutions trained on action recognition datasets can achieve >85% accuracy in classifying athletic movements from video [43,44]. Several studies introduced the Inception architecture with 3D convolutions (I3D), which has been widely applied to action recognition and can be adapted for biomechanical classification tasks [45,46,47,48]. Wang et al. (2016) presented temporal segment networks combining multi-scale 3D convolutions with optical flow processing, achieving state-of-the-art performance in sports action classification [49].

### 5.5. Recurrent Neural Networks and Temporal Modeling

Recurrent neural networks (RNNs) provide an alternative to 3D CNNs for modeling temporal sequences in video data. RNNs process data sequentially, maintaining hidden state variables that represent learned patterns from previous frames. Long Short-Term Memory (LSTM) networks, introduced by Hochreiter and Schmidhuber (1997), address the vanishing gradient problem inherent in standard RNNs through gating mechanisms that control information flow across time steps [50,51,52,53]. LSTMs have been successfully applied to gait phase detection, movement quality classification, and injury risk prediction from continuous kinematic sequences [50,51,52,53].

### 5.6. Transformer Architectures and Attention Mechanisms

Transformer networks, based on the self-attention mechanism, represent a recent paradigm shift in deep learning for sequence modeling. Vaswani et al. (2017) introduced the Transformer architecture, which replaces recurrence entirely with multi-head attention mechanisms that enable parallel processing of sequence elements and capture long-range dependencies more effectively than RNNs [52]. In the context of pose estimation and biomechanical analysis, Transformers enable modeling of complex temporal patterns in athletic movements and inter-joint dependencies (e.g., how hip motion influences knee kinematics) [54,55,56,57,58].

### 5.7. Advanced Keypoint Detection Architectures

Beyond basic pose detection, several specialized architectures have been developed for robust and accurate keypoint (anatomical landmark) identification. Newell et al. (2016) introduced stacked hourglass networks, which employ a symmetric encoder–decoder design with multiple stacked predictions to iteratively refine keypoint localization [59]. Hourglass networks capture multi-scale spatial information and have become standard in pose estimation pipelines. He et al. (2020) presented Mask R-CNN, which extends the popular Faster R-CNN object detection framework to simultaneously detect keypoints and segment body regions, enabling instance-level disambiguation in multi-person scenarios [60]. Sun et al. (2021) introduced HRNet (High-Resolution Network), which maintains high-resolution representations throughout the network via parallel multi-scale branches, achieving state-of-the-art keypoint detection accuracy while remaining computationally efficient [61].

## 6. Force Plate and Pressure-Sensitive Insole Systems

### 6.1. Principles and Technical Specifications

Ground reaction force (GRF) measurement provides quantitative assessment of forces generated at the foot–ground interface during athletic movements. Force plates employ piezoelectric, strain gauge, or capacitive force transducers to measure three-dimensional force vectors (Fx, Fy, Fz) and moments (Mx, My, Mz) at sampling frequencies typically ranging from 500 to 2000 Hz. Standard force plate systems (AMTI, Bertec, Kistler brands) measure ground reaction forces with accuracy better than ±1% of full-scale load [62,63,64]. Force plates provide critical biomechanical data including impact peak forces, loading rates, and center of pressure trajectories that characterize movement quality and injury risk [65].

In field settings where traditional force plates are impractical, pressure-sensitive insole systems provide portable alternatives for quantifying plantar loading during dynamic activities. Two primary commercial systems dominate this market: Pedar-X (Zebris, Germany) and F-Scan (Tekscan, USA).

The Pedar-X system consists of thin, flexible insole elements (approximately 2 mm thick) embedded with 99 capacitive pressure sensors per insole, sampling at 100 Hz with ±5% measurement accuracy and dynamic range from 0 to 900 kPa [66]. Pedar-X insoles are custom-molded to individual feet and worn inside normal athletic footwear, enabling pressure measurement during unrestricted field activities including running, cutting, and sport-specific drills [66,67].

The F-Scan system employs thin resistive film sensors arranged in a 44–66 sensor grid per insole (depending on size), sampling at 100–500 Hz with ±10% accuracy for pressure measurements in the range of 0–1000 kPa [68]. F-Scan insoles are more flexible and thinner than Pedar-X, with minimal impact on foot biomechanics during athletic activities [68].

### 6.2. Validation and Accuracy of Pressure-Sensitive Insoles

Pedar-X and F-Scan systems have been validated against force plate ground reaction forces in multiple studies. Pedar-X specifications report correlation coefficients r = 0.97 for total vertical ground reaction force measured simultaneously on the force plate and insole system [66]. The F-Scan system has demonstrated similar accuracy, with reported correlation coefficients of r = 0.94–0.97 for vertical GRF and root-mean-square error ranging from 5 to 10% of maximum force depending on movement task [68].

Beyond simple force comparison, spatial and temporal characteristics of pressure distribution have been validated. Ranavolo et al. (2020) directly compared Pedar-X insole pressure distributions to force plate center of pressure calculations across multiple walking and running speeds, finding excellent agreement (r > 0.95) in center of pressure location and velocity [69]. Leporace et al. (2020) extended this work to sport-specific movements, validating Pedar-X pressure measurements during jumping, landing, and cutting tasks in soccer players [70].

### 6.3. Clinical Applications in ACL Injury Assessment

Plantar pressure distribution and loading asymmetry between feet are increasingly recognized as functional biomarkers for ACL injury risk and return-to-sport readiness. Abnormal pressure patterns may indicate pain avoidance strategies, proprioceptive deficits, or neuromuscular control insufficiencies following ACL reconstruction [7,17]. Leporace et al. (2020) demonstrated that athletes with persistent loading asymmetry at 6 months post-ACLR (contralateral/ipsilateral peak pressure ratio > 1.15) showed significantly higher reinjury rates during 24-month follow-up [70]. Ranavolo et al. (2020) found that normalized pressure distribution parameters (examining symmetry of peak pressure regions across bilateral limbs) were more sensitive predictors of return-to-sport readiness than traditional strength measures (quadriceps/hamstring LSI) [69].

## 7. Machine Learning and Artificial Intelligence for Biomechanical Classification

Machine learning and artificial intelligence have become integral to automated biomechanical classification and ACL injury risk assessment. Supervised learning architectures, including Long Short-Term Memory (LSTM) networks for temporal kinematic sequence analysis [50], Transformer-based models employing multi-head self-attention for video and pose data [52], and tree-based ensemble methods such as XGBoost and Random Forests for tabular kinematic features [47,71], have demonstrated classification accuracy of 80–92% for distinguishing high-risk from low-risk movement patterns in developmental studies. These approaches enable automated, objective identification of injury-risk kinematics from multi-modal sensor inputs, and form the computational foundation of the AI-driven analysis pipeline integrated into the field-deployable system described in Section 9. A consistent consideration across all deployed clinical AI models is the need for algorithmic transparency and prospective external validation before clinical adoption.

## 8. Clinical Assessment Framework: Three-Tier Approach

### Overview of Risk Stratification and Screening Tiers

Effective ACL injury prevention requires scalable screening methods suitable for diverse clinical and athletic settings with varying resource availability. A three-tier framework progressively increases assessment complexity and cost, enabling initial high-throughput screening followed by targeted advanced testing for at-risk subgroups [7,17,72,73,74]. This tiered approach aligns with public health screening principles and addresses the practical reality that comprehensive laboratory testing of all athletes is cost-prohibitive.

**Tier 1:** Basic Anthropometric and Single-Plane Video Screening: The first tier includes readily deployable measures requiring minimal equipment: height, weight, BMI, limb length measurements, and single-camera video assessment of landing mechanics in the frontal plane. A standard smartphone or tablet camera recording at 60 fps is sufficient for this tier. Tier 1 screening is suitable for large-scale athletic populations (entire teams, school athletic departments) and has minimal time and cost barriers. Visual assessment can be supplemented by manual measurements of knee valgus angle (distance between anterior knee joint line and vertical plumb line), forward trunk lean, and asymmetry observation between limbs [7,17,74]. It is important to note, however, that manual visual estimation of knee valgus from 2D video has well-documented limitations in reliability. Inter-rater reliability for visual knee valgus assessment has been reported to range from poor to moderate (ICC 0.50–0.70) depending on rater experience, viewing angle, and movement task, and intra-rater reliability is similarly variable in untrained assessors. These limitations mean that Tier 1 should be used as a population-level triage tool rather than a definitive risk classification instrument. Standardized rating criteria and rater training protocols are recommended to improve consistency, and athletes identified as borderline or at elevated risk on Tier 1 assessment should be referred to Tier 2 or Tier 3 for objective biomechanical confirmation before clinical decisions are made.
**Tier 2:** Multi-Planar Video and Dual-Task Assessment: Tier 2 screening adds multi-planar video capture (frontal, sagittal, and transverse planes simultaneously via synchronized cameras or single-camera three-quarter views) and introduces attentional demand tasks (dual-task testing combining motor performance with cognitive challenges). Tier 2 assessment can be performed in clinic or field settings with modest equipment investment (2–4 synchronized cameras, ~$500–$2000 total cost). Multi-planar video enables quantification of 3D joint angles through manual digitization or marker-less pose estimation (MediaPipe v0.10, Google LLC, Mountain View, CA, USA; OpenPose v1.7.0, Carnegie Mellon University, Pittsburgh, PA, USA), providing objective kinematic data. Dual-task assessment reveals attention-dependent deficits in movement control, particularly sensitive to neuromuscular deficits in return-to-sport populations [7,17,72,73,74].
**Tier 3:** Comprehensive Multi-Modal Biomechanical Assessment: Tier 3 screening employs comprehensive kinematic analysis combining optical mocap or IMU systems, force plate or pressure-sensitive insole measurement, and integrated machine learning classification. Tier 3 assessment is performed in laboratory or specialized clinic settings and is reserved for: (1) elite athletes in high-injury-risk sports (basketball, soccer, American football, volleyball), (2) post-surgical return-to-sport evaluation following ACLR, (3) athletes with prior ACL injury or family history of ACL injury, or (4) confirmation of elevated risk identified in Tier 1 or 2 screening [7,17,74]. Tier 3 provides comprehensive data suitable for targeted intervention refinement and prospective injury risk prediction. However, the integration of multiple sensor modalities in Tier 3 assessment introduces several practical challenges that warrant acknowledgment [30]. First, temporal synchronization across systems operating at different sampling rates, optical mocap typically at 100–250 Hz, force plates at 1000 Hz, and IMUs at 100–200 Hz, requires hardware trigger systems or software-based alignment algorithms to ensure kinematic and kinetic data are correctly time-stamped. Second, spatial calibration between sensor coordinate systems must be established and maintained throughout the assessment session, as misalignment between systems introduces systematic errors in joint moments and power calculations. Third, real-time data fusion and machine learning classification impose computational demands that may require dedicated processing hardware. Fourth, the setup time, technical expertise, and cost associated with multi-system Tier 3 assessment remain significant barriers to routine clinical implementation. These integration challenges motivate the development of unified, field-deployable platforms that consolidate multiple sensor modalities within a single synchronized system, the approach exemplified by the integrated system described in Section 9.

## 9. Alfayyadh Integrated Drone-Based Motion Capture and Smart Insole System

### 9.1. System Architecture and Components

Disclosure: The system described in this section is the author’s own patented technology (Australian Patent AU2025204327B1), included here as a representative example of integrated field-deployable biomechanical assessment platforms. Performance metrics reported are derived from development-phase testing; independent peer-reviewed validation in prospective athletic cohorts is ongoing and has not yet been published. Readers should interpret the reported metrics accordingly. The Alfayyadh Integrated Drone-Based Motion Capture and Smart Insole System for Real-Time Biomechanical Analysis in Outdoor Environments (Australian Patent AU2025204327B1) represents a novel technological integration designed to translate laboratory-quality biomechanical assessment to field settings [75]. The system comprises four primary components:

Aerial Video Capture Platform: A small unmanned aerial vehicle (sUAV/drone) equipped with a 4K-resolution camera (4096 × 2160 pixels) operating at 60 frames per second, enabling stabilized video acquisition from overhead and oblique angles during outdoor athletic activities [75].

Marker-less Pose Estimation Engine: AI-driven 3D pose reconstruction using deep learning architectures (3D CNNs, LSTMs, Transformer networks) trained on motion capture and sports video datasets, enabling real-time extraction of 3D joint kinematics from drone-acquired video without retroreflective markers [75].

Smart Insole System: Pressure-sensitive insole elements (99 sensors per insole, 100 Hz sampling frequency) integrated with wireless data transmission to a central processing unit, enabling concurrent measurement of plantar pressure distribution and ground reaction force characteristics [75].

Real-Time Data Integration and Analysis Dashboard: A central processing unit combining video-based kinematic data with insole pressure data, performing automated biomechanical assessment against established injury risk thresholds, and generating real-time feedback and risk reports to coaches, athletes, and medical staff [75].

### 9.2. Technical Specifications and Performance Metrics

The 4K video capture at 60 fps provides spatial resolution sufficient for detailed joint-level analysis at typical field distances (15–30 m from subject). At 30 m filming distance with a 4K camera (horizontal field of view ~90°), pixel resolution at subject location is approximately 15–20 mm per pixel, enabling knee joint detection with ±25–30 mm spatial accuracy via marker-less pose estimation, comparable to laboratory standards [75].

Preliminary AI-driven 3D reconstruction metrics have been reported from internal development-phase testing (*n* = 45 athletes; detailed methods in Alfayyadh patent specifications [75]). It is important to note that these figures have not yet been independently validated in peer-reviewed studies and should be interpreted as preliminary benchmarks only. As reported in the patent documentation, the system’s 3D CNN component achieved an area-under-receiver operating characteristic curve (AUC) of 0.82–0.91 in binary classification of high-risk versus low-risk landing mechanics, with 85–89% sensitivity and 78–86% specificity [75]. LSTM temporal analysis achieved 80–92% accuracy in automatic segmentation of movement phases [75], and Transformer network-based classification demonstrated >90% accuracy in binary movement quality classification in developmental cohorts [75]. Independent replication of these metrics in prospective, externally validated cohorts is required before clinical deployment.

### 9.3. Integration of Multi-Modal Data

A key innovation of the Alfayyadh system is concurrent acquisition and integration of multiple biomechanical data streams: (1) video-derived kinematic data (3D joint angles, angular velocities, acceleration patterns), (2) pressure-sensitive insole data (plantar loading patterns, center of pressure trajectories, loading asymmetry metrics), and (3) temporal relationships between kinematic and kinetic data (e.g., correlation between knee valgus onset and peak plantar pressure under medial forefoot regions, which would suggest weight shift toward the medial compartment).

This multi-modal approach enhances the interpretability and validity of injury risk classification. For example, knee valgus alone (kinematic variable) may be confounded by subject anthropometry or individual movement strategies; however, concurrent observation of valgus with loading asymmetry toward the medial foot (kinetic variable) provides stronger evidence of neuromuscular control deficit requiring intervention [75]. It must be acknowledged that the performance metrics reported in this section are derived from development-phase testing and patent documentation rather than independent peer-reviewed validation studies. This represents a significant limitation that constrains the strength of evidence claims that can be made for this system at the present time. Rigorous independent validation would require: (1) prospective cohort studies comparing system outputs against optical mocap and force plate gold standards in independent athlete populations; (2) prospective injury prediction studies with adequate follow-up periods and sample sizes; (3) inter-rater and test–retest reliability assessments under field conditions; and (4) clinical implementation studies evaluating feasibility and screening accuracy in real-world sporting environments. Until such validation data are available in the peer-reviewed literature, the system should be considered a promising developmental platform rather than a clinically validated screening tool.

## 10. Conclusions

Anterior cruciate ligament injury remains a leading cause of morbidity in athletic populations, with 70–80% occurring through modifiable non-contact mechanisms driven by biomechanical risk factors including knee valgus, low knee flexion, tibial internal rotation, and loading asymmetry. Prospective identification of at-risk individuals through biomechanical screening combined with targeted neuromuscular training reduces ACL injury incidence by 50–84%. Advances in computer vision, deep learning, wearable sensors, and integrated field-deployable systems have created unprecedented opportunities for scalable, cost-effective screening that bridges the laboratory–field gap. A practical three-tier framework enables efficient resource allocation from accessible Tier 1 video analysis ($ < 1000) to comprehensive Tier 3 assessment ($ > 100,000), enabling identification and intervention for high-risk subgroups across diverse sporting contexts.

The Alfayyadh Integrated Drone-Based Motion Capture and Smart Insole System exemplifies technological synthesis that provides laboratory-quality biomechanical assessment in ecologically valid field settings through concurrent kinematic and kinetic measurement with AI-driven automated analysis. Future implementation success depends on: (1) prospective validation demonstrating injury prediction accuracy in independent athletic cohorts, (2) standardized protocols enabling cross-platform integration and comparison, (3) long-term randomized controlled trials establishing intervention efficacy, and (4) dissemination strategies ensuring equitable access across resource-limited athletic programs. As evidence of effectiveness accumulates and barriers to accessibility decrease, motion capture and AI-driven biomechanical assessment will increasingly become standard infrastructure in athletic medical care and injury prevention.

## Data Availability

This is a narrative review manuscript. All data cited and analyzed were obtained from publicly available peer-reviewed sources, technical databases, and published literature fully referenced within the manuscript. No original datasets were generated or analyzed during this study.
